# Are synthetic biology standards applicable in everyday research practice?

**DOI:** 10.1111/1751-7915.13612

**Published:** 2020-06-21

**Authors:** Huseyin Tas, Adam Amara, Miguel E. Cueva, Nadine Bongaerts, Alicia Calvo‐Villamañán, Samir Hamadache, Konstantinos Vavitsas

**Affiliations:** ^1^ Systems and Synthetic Biology Program Spanish National Center for Biotechnology (CNB‐CSIC) Madrid Spain; ^2^ Manchester Institute of Biotechnology The University of Manchester Manchester UK; ^3^ SynthSys, CSEC and School of Biological Sciences University of Edinburgh Edinburgh UK; ^4^ Center for Research and Interdisciplinarity (CRI) Université de Paris INSERM U1284 Paris France; ^5^ Synthetic Biology Lab Institut Pasteur Paris France; ^6^ Department of Biochemistry Schulich School of Medicine and Dentistry Western University London ON Canada; ^7^ Department of Biology National and Kapodistrian University of Athens Panepistimioupolis Athens 15784 Greece

## Abstract

The issue of standardization in synthetic biology is a recurring one. As a discipline that incorporates engineering principles into biological designs, synthetic biology needs effective ways to communicate results and allow different researchers (both academic and industrial) to build upon previous results and improve on existing designs. An aspect that is left out of the discussions, especially when they happen at the level of academic and industrial consortia or policymaking, is whether or not standards are applicable or even useful in everyday research practice. In this caucus article, we examine this particular issue with the hope of including it in the standardization discussions agenda and provide insights into a topic that synthetic biology researchers experience daily.

## Graphical abstract

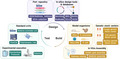

## Introduction

**Fig. 1:**
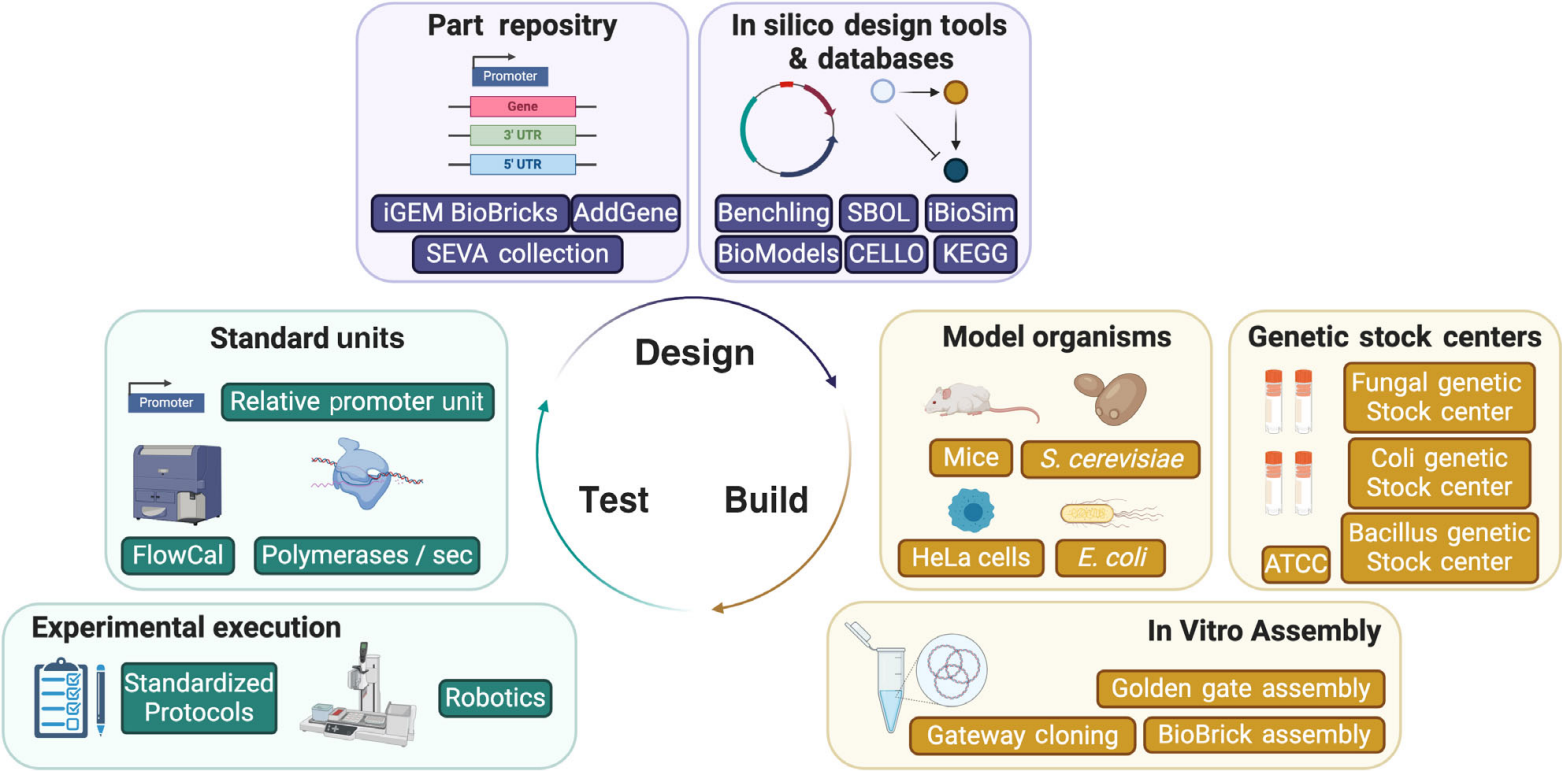
An overview of some of the aspects of synthetic biology where standards could apply.

Synthetic biology, a nascent discipline that aspires to engineer biological systems, is facing a marked standardization challenge. The community recognizes the shortage of universally accepted standards as a problem, with many publications proposing the adoption of specific practices in different areas across the community (Arkin, 2008; Müller and Arndt, 2012; Hillson *et al*., 2016; de Lorenzo and Schmidt, 2018; Hecht *et al*., 2018; Beal *et al*., 2020). While most of the discussion focuses on standards in reporting research outcomes or designs, standards can apply to several aspects of the field (Fig. 1). The debate remains timely, as several ongoing initiatives such as BioRoboost (http://standardsinsynbio.eu/), the Joint Institute for Metrology in Biology (jimb.stanford.edu/sbsc/) and SynbioLEAP (synbioleap.org), have emerged to address the insufficient implementation of standards and provide recommendations to narrow the existing gaps.

Standards are commonplace in many industries, where they facilitate the successful integration, commercialization and scaling up of the serial creation of products or services (Lorenz *et al*., 2019). They help engineers quickly determine whether the behaviour of a device will meet specified requirements. Employing standard practices would open way to better predictions for experimental procedures. In particular relevance to biotechnology, standards allow for accountability and conformity to regulations (such as biosafety and environmental considerations). Consequently, a set of well‐established common standards within synthetic biology can accelerate knowledge transfer and facilitate innovation.

The discussions and initiatives on synthetic biology standardization often emphasize high‐level communication, and reporting between organizations and within the synthetic biology community. However, we think that an essential aspect of standardization, which is the applicability in everyday research, is being overlooked. Can we introduce and embrace a standardized way to perform everyday research tasks? In this article, we examine this question by providing three specific research practices where standards can be adopted, and we discuss the difficulties and drawbacks of introducing standards.

## Standards in everyday research practice

Obviously, not every aspect of research can be standardized, as each research question requires different methodology and adaptability. However, some particular practices lend themselves well to uniformity, allowing for better reproducibility and inter‐laboratory communication. We present here three such examples: *in vitro* and *in vivo* experimental set‐ups, synthetic biology toolbox generation and computational research.

### Case 1: *In vitro* and *in vivo* experiments

Consistency and shared understanding in synthetic biology can be accomplished for both *in vitro* and *in vivo* applications by agreeing on measurement units, tools, protocols, parts, devices and organisms. This standardization will make experimental results more comparable, despite being obtained in different locations by different researchers.

The Kelly standard (Kelly *et al*., 2009) and polymerases per second (PoPS) (Canton *et al*., 2008) approaches are initial attempts towards unit standardization. Other initiatives followed these. For example, the standardization of promoter expressions with the relative promoter unit (RPU) standard aims to normalize promoter strength (Nielsen *et al*., 2016) and FlowCal (Castillo‐Hair *et al*., 2016) came to standardize flow cytometry arbitrary units. However, a practical, universal and comprehensive system of measurement units is still needed. Ideally, standard units should be applicable for or convertible between different chassis organisms without requiring cumbersome experimentation.

In addition to measurement units, experimental protocols themselves can be standardized (i.e. riffyn.com). Standardized protocols enable the comparison of results, especially when applying automation, robotics and artificial intelligence in everyday laboratory practice. The introduction of minimum‐reporting standards (Hecht *et al*., 2018) and the adoption of common best practices become increasingly important.

Most standardization efforts thus far have focused on genetic parts, which are used to assemble genetic circuits that operate differently in varied experimental conditions. Easy access to DNA synthesis will continue to increase our capacity to build genetic constructs, and in turn, the number of parts available, all of which require categorization, naming and standardized reporting. The number of parts and availability of new devices will likely be overwhelming unless the community takes proactive steps to ensure coordinated research, availability, openness and standardized reporting.

Lastly, model organisms are extensively studied organisms with the following common characteristics: easy and relatively inexpensive to gather, transport, maintain and manipulate experimentally (Ankeny and Leonelli, 2011). Although synthetic biology should not restrict itself and forego the breadth and advantages of using a variety of host organisms (Adams, 2016), model species are easier to implement in a standardized set‐up. The transition from ‘model’ to ‘standard’ organism is not automatic. Yet, up to now, notable progress has been accomplished with model organisms – there is recent momentum for the cases of *E. coli, P. putida,* yeast, photosynthetic microorganisms and for plant synthetic biology (openplant.org) – by standardizing community databases, experimental protocols, and stock and strain centres to be in place.

### Case 2: Synthetic biology toolbox generation and adoption

The concept of a toolbox should include every single biological material that is needed in synthetic biology, from DNA fragments themselves to the chassis organisms. This is probably one of the most obvious candidates for standardization, as the tools developed for a particular research approach can benefit the whole community.

Standards are required for the collection and distribution of tools. Cataloguing, depositing and distributing tools under the same platform give access to the detailed history of the tool, while encouraging tool comparison and sharing. Initial attempts of standardizing tools' structure and distribution have a brief but promising history:

Biobricks as a platform for democratizing synthetic biology (parts.igem.org/Help:An_Introduction_to_BioBricks), AddGene as a distribution channel (addgene.org/), SEVA collection as a standard vector collection (seva.cnb.csic.es; Silva‐Rocha *et al*., 2013), OpenMTA for material exchange and transfer agreements (Kahl *et al*., 2018) and a universal Golden Gate standard for photosynthetic organisms (Patron *et al*., 2015; Vavitsas *et al*., 2019) are steps towards standardized cloning practices, making parts developed for different organisms interchangeable.

All the aforementioned examples were the first steps to initiate changes towards the standardization of tools in synthetic biology. However, there is still room for improvement concerning part characterizations and predictable outputs, the completion of which could help to achieve more predictable research outcomes.

### Case 3: Computational standards

Computational biologists face significant standardization challenges due to the incompatibility of computational tools and inconsistent nomenclature (e.g. different names and abbreviations being used for the same metabolite or enzymatic reaction). As one of the purposes of computational work is to enhance the understanding of complex systems, to consider multivariable problems, and to enable automated design, the ability to build upon each other’s work and communicate between different users is crucial.

Computational standards can be categorized into genetic, protein, metabolic, systems‐scale and data‐sharing standards. Many of the standards currently used are derived from the wider biology community. Globally, most of the standards used for computational research come from the fields of systems biology and metabolic engineering (Kohl, 2011), for example genome‐scale metabolic models; the most popular tools and databases (e.g. COBRA, BiGG) (Becker *et al*., 2007; King *et al*., 2016) provide a framework for the standardization of the models and modelling practices. For protein and metabolic applications, well‐known standardization applications are UniProt and KEGG (Kanehisa and Goto, 2000; Apweiler *et al*., 2004). In the case of computational applications, the standards available are mostly focused on the design phase, such as SBOL (Galdzicki *et al*., 2014) for genetic design representation, and are applied in different genetic circuit design tools (e.g. iBioSim, Cello) (Myers *et al*., 2009; Nielsen *et al*., 2016). Most current computational standards do not focus on holistic design, but rather on the partial representation of biological systems (e.g. only genes). This reductionism obliges researchers to use multiple tools – with different and incompatible standards – when working across different complexity levels.

This problem expands in the design, test and learn phases of the synthetic biology cycle. For example, the analysis of omics data to inform subsequent designs can be complicated by the plethora of databases and tools that are not always compatible with each other. Finally, the sharing standards for computational research have been improved by different platforms that standardize data and models sharing.

## New standards introduction, adoption and challenges

Many widely used standards can come from leading organizations or companies developing products and practices widely adopted by the community. A popular example is the TrueType computer font standard developed in the ’90s by Apple, and adopted by Microsoft, quickly becoming a standard in the industry. However, these types of standards are not necessarily the best ones as they are developed for a defined industrial product, but they impose themselves when no other solutions exist.

This brings us to the next question. What are the limits we should impose on standardization?

There are a few instances where strict standardization might be of little benefit or even a hindrance. One example is the BioBricks standard for genetic assembly, which was revolutionary when first appeared, but became disused as more efficient cloning methods became available. Until the 2019 competition, iGEM required the use of BioBricks format for valid participation. As a result, teams that made their normal constructs using isothermal assembly or Golden Gate faced the extra burden of making BioBricks‐compatible genetic parts solely for ticking off the medal criteria. This is a good example of why standards should not be something static, but rather evolve to correspond to the best available practices. Otherwise, they create unnecessary workload or prevent out‐of‐the‐box thinking, thus stifling innovation.

Synthetic biology, as an interdisciplinary field, applies diverse techniques from biology (with its many subfields), chemistry, mathematics, computer science, engineering and physics, and each of these areas comes with its own researchers, practices and standards (Krohs and Bedau, 2013). So a major challenge is developing adaptive standards and frameworks for synthetic biology, ones that will allow and are capable of harmoniously integrating components that use external standards. Standards for systems integration are required to build complex biological systems, just like integrated systems design in aerospace enables different parts complying with different standards to work together efficiently (Monell and Piland, 2000).

## Conclusion

Synthetic biology is handling the transition from a traditional molecular biology paradigm, to quantifiable, well‐defined, industrialized and production‐based scientific approaches. In this caucus article, we emphasize on researchers' daily experiences and the applicability of standards in everyday laboratory practice. We believe that yes, standards are applicable in everyday laboratory practice, and the three cases we address corroborate this line of thinking. By standardizing, synthetic biology will gain numerous advantages and will better meet the industry’s demands of strict predictability. But we need to apply standardization with a degree of caution and use standards as a tool, not as the end goal. The specification and adoption of standards should take into account the needs of the community in a comprehensive and flexible manner. We, the synthetic biology community, should be aware that any designs and tools have limitations. And standards cannot be easily implemented unless we come up with user‐friendly and Internet‐friendly collections and distribution platforms – ideally maintained by governmental support and dedicated international grants and consortiums that include the industry.

## Conflicts of interest

The authors declare no conflict of interest.
